# The Toolbox for Untangling Chromosome Architecture in Immune Cells

**DOI:** 10.3389/fimmu.2021.670884

**Published:** 2021-04-29

**Authors:** Shuai Liu, Keji Zhao

**Affiliations:** Laboratory of Epigenome Biology, Systems Biology Center, NHLBI, NIH, Bethesda, MD, United States

**Keywords:** three-dimensional chromatin organization, enhancer-promoter interactions, transcriptional regulation, epigenome, cell differentiation

## Abstract

The code of life is not only encrypted in the sequence of DNA but also in the way it is organized into chromosomes. Chromosome architecture is gradually being recognized as an important player in regulating cell activities (e.g., controlling spatiotemporal gene expression). In the past decade, the toolbox for elucidating genome structure has been expanding, providing an opportunity to explore this under charted territory. In this review, we will introduce the recent advancements in approaches for mapping spatial organization of the genome, emphasizing applications of these techniques to immune cells, and trying to bridge chromosome structure with immune cell activities.

## Conflict of Interest

The authors declare that the research was conducted in the absence of any commercial or financial relationships that could be construed as a potential conflict of interest.

## Introduction

The spatial-temporal gene expression determines the identity and activity of cells (1, 2). Gene expression is controlled by transcription factors working on the dynamically structured genome (3–6). Elucidating genome architecture is pivotal for understanding the fundamental mechanisms of gene expression regulation. Thanks to technological developments, our understanding of genome structure has been revolutionized in the past decade (7–10).

Eukaryotic genomes are organized into chromatin with nucleosomes as fundamental structural units (11). The spatial organization of chromatin in the nucleus has been investigated at several scales. First, formation of chromatin loops brings distant genomic regions from tens to hundreds of kilobases to spatial proximity, which involves the Cohesin complex through an extrusion process (12–17). The next level of chromatin organization is topologically associating domains (TADs), ranging from hundreds of kilobases to several million bases (18–20). The boundaries of TADs are usually marked by the binding of insulator CCCTC-binding factor (CTCF), which plays a key role in the formation of TADs (20–22). TAD boundaries appear to restrict chromatin loops within TADs and few chromatin loops form across TAD boundaries (20, 23). Chromatin can also be separated into even larger domains, termed as A compartment and B compartment, which are enriched for active and repressive chromatin modification marks, respectively (23, 24). A compartment is composed of active TADs, which often contain expressed genes, while B compartments contain silent genes. Each chromosome occupies a specific location in the 3D nuclear space, which is termed as chromosome territory (24, 25). From nucleosome to chromosome territory, the architecture of chromatin features a hierarchical pattern.

From a methodology perspective, RNA-seq method provides transcriptome information (26), and ChIP-seq are used for investigating transcription factor binding and histone modification profiles on the genome (27, 28). Techniques for mapping the landscape of chromatin accessibility include ATAC-seq and DNase-seq (29). The techniques for cracking the three-dimensional genome architecture have been rapidly evolving during the last decade. In this review, we will introduce current state-of-art techniques for untangling the three-dimensional (3D) organization of chromatin, and then discuss their applications to the immune system. Two classes of approaches are extensively used in exploring the 3D genome organization: microscopy-imaging based techniques and sequencing based methods (**Table 1**). We will briefly introduce imaging-based methods, and then focus on sequencing-based approaches.

**Table 1:** Comparison of the features of techniques for elucidating chromatin structures.

Methods		Number of cells	Number of contacts	Advantages	Shortcomings	References
**Imaging**	2D/3D FISH	Several hundreds	Two to tens of loci	Spatial distance between loci in single cell	Low resolution, low throughput	(30–33)
	Multiplexed FISH and STORM	several hundreds to thousands	Over 1,000 loci	Higher resolution and throughput comparing to conventional FISH, spatial organization in single cell	Laborious, low throughput comparing to sequencing based methods	(34–38)
	Live-cell imaging	Several hundreds	Two to tens of loci	Visualize dynamics of chromatin structure in single cell	Low resolution, low throughput	(39–41)
**3C based**	3C	100 million	One *versus* one	Easy to perform experiments and analyze data	Only for contacts between two target locus	(42, 43)
	4C	1–10 million	One *versus* all	Discover new contact partners	Only detects pairwise contacts	(44, 45)
	5C	2–5 million	Many *versus* many	Simultaneously detects contacts between many locus of interest	Requires information of the locus, resolution and throughput relies on the number of probes	(46, 47)
	Hi-C	1–20 million	All *versus* all	Whole genome organization map	High sequencing depth required for high resolution, detects only pairwise interactions	(24, 48)
	capture Hi-C	1–20 million	Many *versus* all	Detect interactions of selected loci	Requires specifically designed probes	(49–51)
	ChIA-PET	100 million	Many *versus* many	Works on interactions related with a protein target	Requires large cell number, need high quality antibody	(52, 53)
	Hi-ChIP	50,000–25 million	Many *versus* many	Works on interactions related with a protein target, small cell number	Requires high quality antibody	(54, 55)
	Micro-C	1,000–5 million	All *versus* all	High resolution	Not good for capturing long-range interactions	(56, 57)
**Proximity ligation free**	GAM	Several hundreds	All *versus* all	No proximity ligation bias, captures long-range interactions, provide spatial organization information in single cell	Requires special equipment, low resolution	(58)
	SPRITE	10 million	All *versus* all	No proximity ligation bias, captures multi-way interactions	Requires efficient multiple rounds of index ligation	(59, 60)
	ChIA-Drop	10 million	All *versus* all	No proximity ligation bias, captures multi-way interactions	Requires a 10X Genomics sequencing platform	(61)
	DamC	10,000–1 million	One *versus* all	Detect *in vivo* contacts	Need to manipulate the cells to express the fusion protein of Dam and the target of interest	(62)
	TrAC-looping	50 million	Many *versus* many	No proximity ligation bias, provides chromatin accessibility information	Requires large cell number, only detects interactions in open chromatin regions	(63)

## Imaging-Based Techniques

Fluorescence *in situ* hybridization (FISH) was once the dominating method for studying genome structure (30, 31, 64, 65). The spatial distance between chromatin loci is visualized under the microscope by hybridizing fluorescently labeled probes to target regions in fixed cells. DNA-FISH is suitable for detecting the spatial distances between two or a few loci. However, the two major limitations, resolution and throughput, hamper its applications. The resolution of FISH was constrained by microscopy and the probe, making it not suitable of resolving loci within relatively close spatial distance (shorter than several hundred nanometers) or close locations on the genome (less than 100 kb). Recently, there have been significant improvements in the resolution and throughput of FISH microscopy with the development of short multiplexed probes and super-resolution microscopy (32–36, 66). Genome architecture in single cells can now be visualized at spatial resolution of tens of nanometers and genome resolution of tens of kilobase pairs (36, 37, 67, 68). One major advantage of imaging based techniques is that they are capable of monitoring the dynamics of chromatin structure in living cells (38, 69), which was reviewed recently (39). Even though the resolution of these approaches is relatively low, they still provide invaluable knowledge about the dynamics of genome architecture. It would be of interest to observe how the genome architecture of immune cells changes in response to environmental stimulus. Fluorescence and electron imaging techniques are evolving rapidly for elucidating chromatin organization. There are excellent reviews on this topic, which will not be discussed in detail here (40, 41, 70, 71). Although these cutting-edge imaging techniques have not been widely applied to studying the immune system, with the improvement of resolution and throughput, they will be sure to illuminate the connection between genome organization and immune cell activity.

## Sequencing-Based Techniques

**Figure 1:**
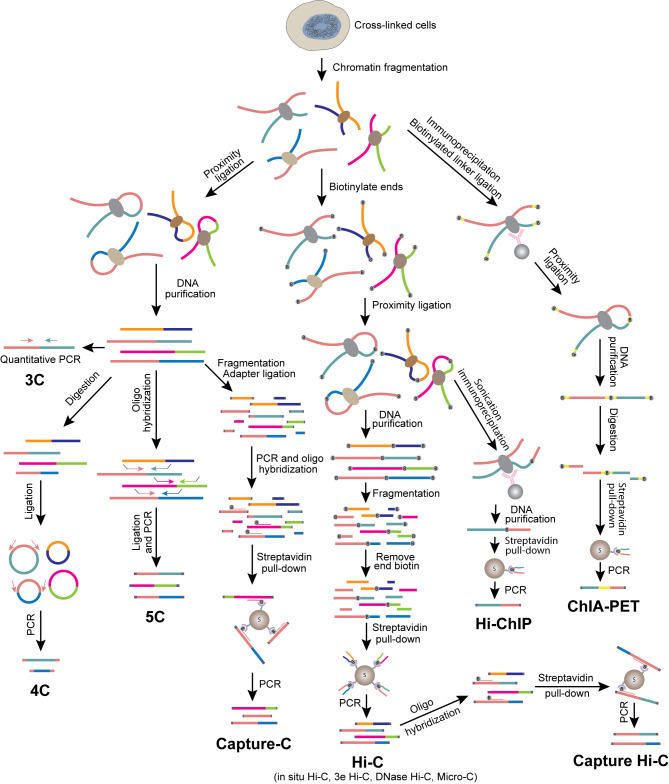
Overview of 3C-based methods. Schematics illustrate experimental procedures of different methods.

In our discussion, sequencing based approaches for mapping genome architecture are separated into two categories: proximity ligation-based methods (**Figure 1**) and proximity ligation-free methods.

### Proximity Ligation-Based Methods

#### (1) Chromosome Conformation Capture (3C)

Unlike FISH directly presenting the physical distance between genomic loci, chromosome conformation capture assay (3C) detects the spatial proximity between two genomic loci by interpreting the efficiency of them being ligated together (72, 73). Cells are fixed with formaldehyde to preserve the spatial conformation of chromatin, and chromatin is then digested with a restriction enzyme to generate free ends of DNA. The DNA ends from chromatin regions in close spatial proximity are prone to be ligated in the presence of DNA ligase. The frequency of ligation between two loci can be examined by gel electrophoresis or qPCR using primers specific to these regions. 3C detects the interaction between two candidate loci (one-to-one). FISH and 3C are frequently used together to cross-validate each other’s findings. Even with various more advanced methods available as discussed below, 3C remains a good choice if one is interested in examining the changes of interaction between two specific genomic regions, especially transcriptional regulatory regions, during cell differentiation or activation.

#### (2) Circular Chromosome Conformation Capture (4C) and Chromosome Conformation Capture Carbon Copy (5C)

4C was developed to identify genome-wide interacting partners of a target locus (the “viewpoint”) (74, 75). Unlike 3C, which requires knowledge of the interacting locus, 4C is capable of discovering unknown interactions. The 4C protocol requires a DNA circularization step after proximity ligation, followed by reverse PCR with primers designed according to the sequence of the viewpoint. All interacting partners can then be identified by sequencing. Thus, 4C is a method for detecting one-to-all interactions. By comparison, 5C is a method for detecting many-to-many interactions. Since all interacting partners are supposed to be ligated at the proximity ligation step in 3C, multiple interacting pairs may be amplified and detected by using properly designed multiplexed primers in a 5C library. The coverage and resolution of 5C depends on the diversity of the primers (76, 77).

#### (3) Hi-C and Derivatives

In the past decade, a major breakthrough in the field of elucidating genome architecture was the development of Hi-C, a method for analyzing whole genome organization (all-to-all interactions) (24). Hi-C follows the original 3C protocol with some modifications including addition of a biotinylated nucleotide fill-in step before proximity ligation. To generate Hi-C libraries, biotin-labeled and ligated DNAs are enriched, digested and ligated to Y-shaped adaptors for next generation sequencing (NGS). After sequencing on an NGS platform and mapping the reads to the genome, a genome-wide contact map can be established from the paired-end sequencing reads. Hi-C has provided tremendous information about genome organization, from compartments and TADs to chromatin loops. Efforts have been made to optimize Hi-C to generate higher resolution genome organization map (78). In situ Hi-C decreases random ligation between chromatin fragments due to reduced nucleus disruption (23, 42). The resolution of a genome-wide chromatin contact map generated by Hi-C is limited by the available restriction enzyme cleavage sites in the genome. Thus, digesting the chromatin by using a combination of restriction enzymes (3e Hi-C) (43), DNase I (DNase Hi-C) (44) or micrococcal nuclease (Micro-C) significantly increases the resolution (45, 46). Application of Hi-C to immune cells has revealed cell-specific three-dimensional chromatin interactions and provided insights into the mechanisms of their regulation in immune cells (43, 47).

Although Hi-C has the advantage of being capable of providing genome-wide chromatin contact maps, it suffers from the need for very deep sequencing in order to obtain information on chromatin interaction among transcriptional regulatory elements such as promoters and enhancers. The cost of sequencing to examine the fine chromatin structures is often prohibitory (23, 24, 48). Only a very limited number of interactions between promoters and enhancers are detected even with a sequencing depth of billions of PETs from a Hi-C library. To increase the resolution and efficiency and reduce the sequencing cost of Hi-C, a few derivative approaches were developed. One strategy is the Capture Hi-C method that detects chromatin contacts of selected chromatin regions such as gene promoters (56, 79–81). After proximity ligations, genomic loci of interest are captured and enriched by hybridizing biotinylated oligonucleotide probes. This strategy provides the chromatin interaction information of the selected genomic regions. ChIA-PET (Chromatin Interaction Analysis with Paired-End Tag) and HiChIP are strategies of detecting chromatin interactions of a subset of genomic regions by combining Hi-C and ChIP (chromatin immunoprecipitation) (49, 57, 82–84). ChIA-PET performs proximity ligation after the ChIP step by using a specific antibody against a transcription factor (TF), chromatin modifier or histone modification to enrich target regions, while HiChIP performs ChIP after the proximity ligation step. These two approaches are capable of profiling chromatin interactions genome-wide at locations bound by a specific chromatin protein or carrying a specific histone modification. For example, the promoter-enhancer interaction network explored by RNA Pol II ChIA-PET in GM12878 cells provided comprehensive view of the regulation of B cell transformation triggered by Epstein-Barr Virus (EBV) infection (50, 51).

Hi-C is a powerful technique for detecting chromatin interactions; however, the heterogeneity of chromatin structure in each individual cell is concealed in this population averaged approach. In situ single-cell Hi-C and Dip-C are two methods for exploring the diversity of genome organization at a single cell level (52–55). Nagano et al. applied single-cell Hi-C to tracking the dynamics of chromatin structure in cell cycle at a single cell level (85). Flyamer et al. discovered reorganization of chromatin in the transition of oocyte to zygote (86). By using Dip-C, Tan et al. investigated genome organization in neurons of mouse retina, olfactory epithelium and developing brain (87, 88). By applying these techniques to immune cells, it would reveal interesting diversity features of genome architecture at a single cell level, such as the recombination of antigen receptors.

When performing 3C experiments, chromatin conformation is normally preserved by fixing cells with formaldehyde. However, chemical crosslinking may introduce bias in detecting chromatin interactions. Intrinsic 3C/4C/Hi-C (i3C/i4C/iHi-C) and liquid chromatin Hi-C are developed to study native genome structure (89, 90). i3C captures chromatin structures detected by conventional 3C with lower background. Liquid chromatin Hi-C is capable of tracking dynamics of chromatin interactions. By using i4C, Weiterer et al. studied chromatin structures at *CXCL2* and *IL8* loci with the stimulation of interleukin (IL)-1α, and found that IL-1α–induced chromatin remodeling depends on TAK1 kinase and NF-κB pathways (91).

#### (4) Other Proximity Ligation-Based Methods

Conventional 3C techniques reconstruct genome architecture based on averaged pairwise chromatin interactions. It remains to be elucidated how these interaction pairs are synergized into higher order structures. Recently developed chromosomal walks (C-walks), multi-contact 4C (MC-4C), Tri-C, multi-contact 3C (MC-3C) and other methods provide insights into concurrent chromatin interactions at single allele levels (92–97).

Majority of noncoding RNAs (ncRNAs) localize in the nucleus, and have contacts with chromatin. They play important roles in gene regulation and chromatin remodeling (98). Proximity ligation-based methods, such as MARGI (mapping RNA-genome interactions), GRID-seq (global RNA interactions with DNA by deep-sequencing), ChAR-seq (chromatin-associated RNA sequencing) and RADICL-seq (RNA and DNA interacting complexes ligated and sequenced), were developed to detect genome-wide RNA-chromatin contacts (99–102). They share a similar approach by bridging interacting RNA and DNA with a bivalent linker.

### Proximity Ligation-Free Approaches

All 3C-derived methods require proximity-based ligation. They identify ligation frequency between loci instead of direct physical contact information. Since the products are generated by pair-wise ligation between different genomic regions, these methods are not effective at capturing multiple contact partners of a locus simultaneously. Furthermore, artifacts could also be generated by the proximity ligation step. To address these caveats, proximity ligation-free techniques have been emerging.

#### (1) Genome Architecture Mapping (GAM)

GAM is performed by cryo-sectioning fixed and sucrose-embedded nuclei (103). Hundreds of nuclei are sectioned in random orientations. DNA from each slice is extracted, indexed and sequenced. Chromatin contact information can be inferred from the sequencing data by counting the chance of DNA loci co-existing in the same slice. A mathematical model named SLICE (statistical inference of co-segregation) was developed for analyzing GAM data. GAM reveals long-distance chromatin loops and TADs structure similar to that from Hi-C analysis but at the single-cell level. However, due to the limited number of slices that can be generated from each nucleus, the method suffers from low resolution, particularly for studies of promoter-enhancer interactions.

#### (2) Split-Pool Recognition of Interactions by Tag Extension (SPRITE) and ChIA-Drop

Chromatin complexes are formed by chromatin binding proteins and their target DNA, which could be stabilized by formaldehyde crosslinking. The interacting chromatin regions that remain in the same complex after cleavage of chromatin will be labeled by the same set of barcoding indexes after multiple of rounds of index ligation. Chromatin regions, which are in different chromatin complexes, are labeled by different barcoding indexes in this process. Based on this property, SPRITE and ChIA-drop assign chromatin regions to different complexes, which infers the spatial proximity and thus potential interaction between different regions of chromatin (104–106). Tagging in SPRITE is performed by ligation in multiple rounds of split-pool, whereas, the reaction adding barcoding indexes to each complex in ChIA-drop takes place in a droplet. DNA loci carrying the same barcode are considered to be in the same interacting complex. Multiple chromatin regions could be assigned to the same chromatin complex, which implies multiple ways of interactions in addition to pairwise interactions.

#### (3) DamC

DamC uses *Escherichia coli* DNA adenine methyltransferase (Dam) fused with rTetR, which binds to TetOs inserted into the genome in the presence of doxycycline (Dox) (107). The bound rTetR-Dam methylate adenines in GATC sequences near its binding sites or regions in spatial proximity. Methylated locations are identified by DpnI, which specifically cleaves adenine methylated GATC sequences in the genome. The cleavage sites are ligated to sequencing adapters and analyzed by sequencing. This method generates 4C-like data (one-to-all) with a viewpoint of TetOs that are inserted into the genome. Data of many-to-all interactions can be generated using cell lines with multiple sites of TetOs in the genome. DamC provides genome structure information in living cells; however, it requires manipulating the genome to insert “viewpoints” and to express rTetR-Dam protein in the cells of interest.

#### (4) Transposase-Mediated Analysis of Chromatin Looping (TrAC-Looping)

Our lab developed a method termed as TrAC-looping (transposase-mediated analysis of chromatin looping), which detects chromatin interactions at transposase Tn5 accessible regions (108). This method relies on Tn5’s ability to insert DNA into the genome. Tn5 forms a tetramer complex with a specially designed bivalent mosaic ends (ME) oligonucleotide linker, which inserts one ME end of the bivalent linker to a chromatin region and the other ME end to an interacting chromatin region. By this way, the two interacting regions are directly linked by an oligonucleotide bridge and thus can be amplified by PCR as one DNA template using primers recognizing the ME sequence and oligonucleotide linker. TrAC-looping detects all interactions between transcription regulatory regions, which include all active promoters and enhancers. TrAC-looping data simultaneously inform both chromatin accessibility and chromatin interactions. Only modest sequencing depth is required for measuring genome-wide regulatory chromatin interactions at high resolution.

## Profiling Genome Organization in Immune Cell Development and Differentiation

**Figure 2:**
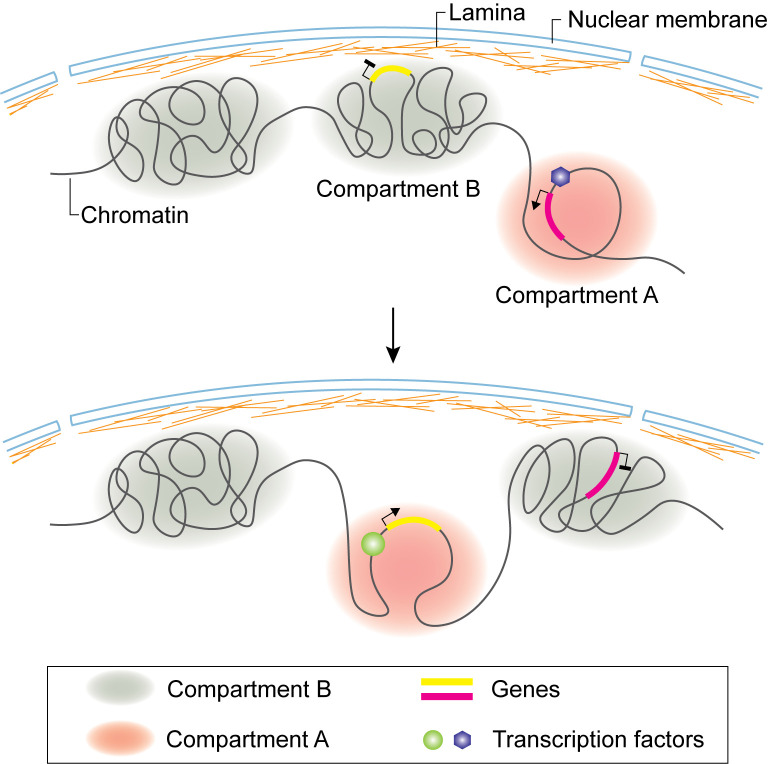
Compartment switch in B cell and T cell lineage commitment. Loci of B or T cell lineage specific genes dissociate from nuclear lamina and are flipped from inactive B compartment to active A compartment. Compartment switch is accompanied by expression of specific transcription factors regulating target gene expression.

Next, we will discuss recent applications of these various methods to investigate the genome architecture in immune cells during development and differentiation. The development and differentiation of immune cells are accompanied by reorganization of the genome architecture, from compartment switch to the formation of loops between regulatory elements (**Figure 2**). Tissue microenvironment triggers the transformation of genome structure, producing key transcription factors (TFs), which regulate the expression of lineage specific genes, determining the fate and function of the cells. The genome organization during immune cell development and differentiation, especially during lineage commitment and antigen receptor *V(D)J* recombination, has been extensively studied by various approaches.

### Genome Architecture of B Cells

B cell development takes place in bone marrow. Early B cell factor (EBF1) is one of the key transcription factors that control the commitment of progenitor cells to B-cell lineage. By combining Hi-C and 3D-FISH, it was observed that the *Ebf1*gene locus is sequestered at the nuclear lamina as a repressive B compartment in progenitor cells. When the progenitor cells transitioned to the pro-B stage, the *Ebf1* locus switched to an active A compartment, with the establishment of stage specific new interactions (**Figure 2**) (109). Transcription factor Paired box 5 (PAX5) locks B-cell commitment by regulating the expression of lineage specific genes. By using Hi-C, it was found that PAX5 plays a critical role in establishing and maintaining the unique genome structural features in B cells throughout the B cell differentiation process (58). Ultimately, it regulates gene expression by rewiring the interactions between enhancers and promoters (58).

**Figure 3:**
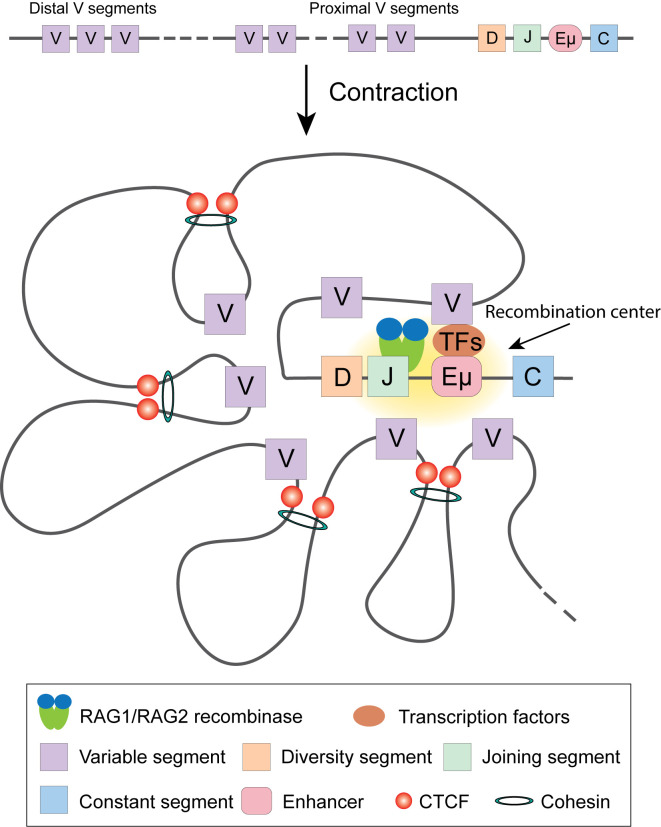
V(D)J recombination of *Igh* locus. Transcription factors target Enhancer µ and recruit RAG1/RAG2 recombinase to execute recombination. In committed pro-B cells, Cohesin complex and CTCF facilitate *Igh* locus contraction by looping, ensuring each V gene segment has equal opportunity to target DJ segment.

To fight against various antigens, the adaptive immune system depends on B and T cells producing a diverse pool of antigen specific receptors, immunoglobulins and T cell receptors (TCRs), respectively, through the process of *V(D)J* gene recombination (**Figure 3**) (59). The recombination of immunoglobulin heavy chain (*Igh*) and light chain (*Igl*) takes place in Pro-B and Small pre-B stages, respectively. The initiation of recombination is carried out by RAG recombinases, which are expressed in developing T and B cells. A Hi-C study revealed that the expression of RAGs is regulated by transcription factor E2A, which facilitates the formation of chromatin loops between their promoters with the T cell-specific enhancer (*R-TEn*) or B cell-specific elements (*R1B* and *R2B*) *(* 60). During the transition from progenitor cells to pro-B cells, by using 3D FISH, it was observed that the *Igh* locus is released from the nuclear periphery and undergoes chromatin decompaction, suggesting that nuclear periphery localization represses the transcription and recombination of *Ig* genes during lymphocyte development (61). It was also visualized by FISH that distal *Igh V* regions are brought into close proximity of the *DJ* cluster to generate diverse Igh populations (62, 63), which is mediated by the formation of chromatin looping (110). By combining FISH and 3C, Guo et al. discovered that the *Igh* locus folds into several multi-looped domains, which are subsequently brought into proximity for selective recombination of the enhancer Eµ dependent looping with specific sites within *V_H_* region (111).

Close inspection of *Igh* recombination in live pro-B cells revealed that the contact between *V_H_* and *D_H_J_H_* loci happens in minutes and the interaction is controlled by the spatial confinement from the formation of topological domains (112). Using 4C, Medvedovic et al. extensively studied the organization of the *Igh* locus from different viewpoints (113). They found that distal *V_H_* cluster showed more flexibility than the proximal domain, ensuring equal chance of distal *V_H_* to recombine with D_H_J_H_. The long-range looping requires regulators PAX5, YY1, and CTCF. Convincing data indicated the important roles of CTCF and Cohesin in organizing chromatin loops at the *Igh* locus and their involvement in *Igh* recombination (114–117). Cohesin complex facilitates RAG endonuclease scanning along the chromatin fiber by loop extrusion, which initiates immunoglobulin recombination. CTCF bound at its target sites stabilizes the contact between RAG and the distal *V_H_* locus, promoting the recombination to happen (17, 110, 118–120). PAX5 specifically represses the expression of Cohesin release factor WAPl to ensure successful loop extrusion across the entire *Igh* locus (121).

Another major chromatin reorganization event takes place upon the activation of B cells. To elucidate the cis-regulomes in activated B cells, Chaudhri et al. studied the interaction network of cis-regulatory elements by Hi-C. They discovered multiplex enhancer-promoter interaction configurations, including genes regulated by multiple enhancers and enhancers controlling multiple genes, which are engaged in B cell activation, cycling and differentiation (122).

### Genome Architecture of T Cells

Multipotent hematopoietic progenitor cells migrate into the thymus, then initiate differentiation from double negative (DN, CD4^−^ CD8^−^) to double positive (DP, CD4^+^ CD8^+^) and finally the CD4 or CD8 single positive T cells. DN cells can be further separated into several stages from ETP, DN2, DN3 to DN4. While ETP and DN2 cells can take alternative cell fates such as dendritic cells, NK cells and mast cells, DN3 cells are already committed to T cell lineage. Thus, the transition from DN2 to DN3 is a key step of T cell fate commitment. Previous studies identified BCL11B, which is upregulated at the DN2 stage, as a key transcription factor required for DN2 to DN3 transition (123–125). Hu et al. used Hi-C to survey the dynamic changes of genome architecture from HSCs to DP cells and observed a striking reorganization of chromatin during the transition from DN2 to DN3 (47). While all other transitions display a relatively smooth change in chromatin interaction, the DN2 to DN3 transition is accompanied by an abrupt global transformation of the 3D genome structure, suggesting that the global changes in chromatin organization may lock the cells in the T cell lineage by creating an energy barrier for reversing cell fate. This observation led to the question of what process causes this global reorganization of chromatin? BCL11B was found to bind to numerous key regulatory regions accompanying T cell commitment (47, 124). Interestingly, BCL11B is a core subunit of the ATP-dependent chromatin remodeling BAF complexes, suggesting that it may be a key player in promoting the global transformation of chromatin reorganization during the DN2 to DN3 transition. Although it has not been demonstrated yet whether BCL11B regulates the changes of chromatin interaction during the early development of T progenitor cells, its deletion in CD4 T cells resulted in decreased chromatin interaction at its bound regions (47). The *Bcl11b* locus is also associated with a switch from the repressive B compartment to active A compartment when BCL11B expression is upregulated at the DN2 stage. DNA-FISH confirmed the locus moves away from the repressive nuclear periphery during this process. This work demonstrated that, during T cell differentiation, remodeling of chromatin landscape initiates the expression of key transcription factors, which reorganize chromatin structure and alter gene expression to determine the fate of the cells. The activation of *Bcl11b* is mediated by a non-coding RNA ThymoD (thymocyte differentiation factor). Combining Hi-C and DNA-FISH, Isoda et al. found that ThymoD promotes BCL11B expression by facilitating the formation of chromatin looping between its enhancer and promoter mediated by CTCF and Cohesin (126).

Similar to the recombination of immunoglobulin loci in B cell development, reorganization of T cell antigen receptors also takes place during T cell development. Based on the composition of T cell receptors (TCRs), T cells are separated into Tαβ and Tγδ lineages. The rearrangement of β, γ and δ TCRs takes place in DN cells, whereas TCRα recombination takes place in DP cells. Lineage-specific recombination of TCR is partially determined by the cell specific expression of RAG1 and RAG2. Hi-C analysis revealed that there is a unique organization pattern of cis-regulatory elements in developing T cells (60). Transcription factor E2A binds to a T cell lineage-specific enhancer (*R-TEn*) to orchestrate the chromatin conformation and expression of the *Rag* genes in T cells. The chromatin organization landscape of the *Tcr* locus changes in a spatial and temporal pattern during T cell development. A DamID study identified a lamina-associated domain (LAD), which represses the recombination and expression of *Tcrb (* 127). In DN cells, LAD disassociates from lamina, and *Tcrb* adopts a compact conformation, enabling the recombination between *Vβ* and *DβJβ* segments (128). 3C and 4C analysis indicated that the distal *Vβ* segments are separated from the recombination center in DP cells (129). Enhancer *Eβ* is important for the expression of *Tcrb*. Zhao et al. studied the looping between *Eβ* and *Tcrb* promoter by 3C (130). They found transcription factor RUNX1 facilitates the rearrangement of chromatin loops. Different transcription factors may contribute to the rearrangement of chromatin interaction landscape of different *Tcr* loci. For example, STAT5 binding to *Jγ* promoter is essential for the change of chromatin conformation at the *Tcrg* locus (131). A series of work, by combining 3C, 4C, Hi-C and DNA-FISH, have demonstrated that chromatin looping organized by CTCF and Cohesin plays an important role in shaping the diversity of TCR repertoire (117, 132–136).

Mature naïve CD4 or CD8 single positive (SP) cells exit the thymus. They are activated upon encountering antigens and differentiate to effector or memory cells. This process is associated with global epigenomic changes (137). Three-dimensional chromatin organization has been extensively studied in these cells by different approaches, especially for CD4^+^ T helper (Th) cells. Our lab looked into the genome-wide promoter-enhancer interaction in naïve and activated human CD4^+^ T cells by TrAC-looping and ChIA-PET (84, 108). It was found that genes with promoters which have interacting enhancers are expressed at higher levels than those without interacting enhancers, and their expression levels are positively correlated with the number of interacting enhancers. A large number of promoters interact with other promoters and they are co-expressed in a tissue-specific manner (84, 108). Activation of naïve CD4^+^ T cells is associated with significant changes of chromatin interaction at thousands of promoters and enhancers, which are correlated with the changes in gene expression (84, 108). The FOS family motif, TGAGTCA, was significantly enriched in chromatin regions with increased interaction and ChIP-seq assays confirmed that the binding of FOSL1/Fra-1 to these regions after T cell activation. These data suggest that the binding of the FOS family factors may contribute to the increased interactions.

**Figure 4:**
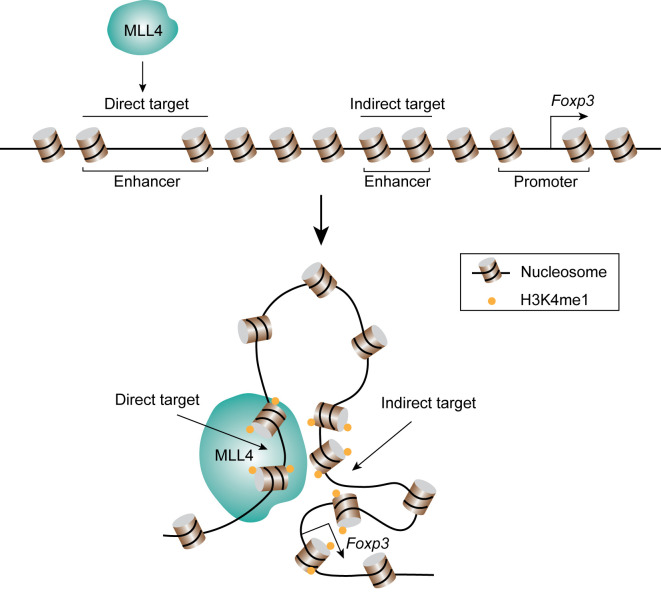
MLL4 catalyzes H3K4 methylation through chromatin looping. MLL4 binds its direct target region, and the lysine 4 of histone H3 at distal indirect target regions are also methylated through chromatin looping.

Naïve CD4^+^ cells differentiate into Th1, Th2, Treg, Th17, or Tfh effector cells by the expression of key transcription factors. T-bet is the key transcription factor for Th1 differentiation and the expression of cytokine interferon-γ (IFNG). DNA-FISH combined with 3C analysis indicated that T-bet collaborates with CTCF and Cohesin to facilitate the formation of chromatin loops between the regulatory elements at *Ifng* gene locus and promote its expression (138, 139). The transcription factor GATA3 critically regulates Th2 differentiation and the expression of Th2 cytokines, IL4, IL13, and IL5. It was found that the Th2 locus control regions (LCR) contributes to the expression of Th2 cytokines through the formation of long-range chromatin loops with the promoter regions (140). GATA3 collaborates with STAT6 and YY1 to regulate the chromatin interaction of the Th2 cytokine locus (141, 142). Interestingly, interchromosomal interactions between Th2 LCR (on chromosome 11) and the promoter of *Ifng* (on chromosome 10) or *IL-17* (on chromosome 1) were discovered by 3C and FISH analysis (143, 144). These results suggest long-range chromatin interactions between functionally important genes may play a role in coordinating the expression of key cytokines in the T cell fate decision. The basic leucine zipper transcription factor, BATF, is one of the key transcription factors for the differentiation of Th17 and Tfh cells. A recent study using Hi-C and 3C assays demonstrated that BATF, cooperating with ETS1, recruits CTCF to lineage-specific genes and reorganizes chromatin landscapes in developing effector cells (145). By using a combination of ChIP-seq, Hi-C and CRISPR-mediated genomic editing, our lab discovered a novel transcriptional regulatory element about 8.5 kb of upstream of the *Foxp3* gene promoter for the expression of FOXP3 in Treg cells (146). The data revealed that this element interacts with the *Foxp3* promoter and intronic enhancers and its deletion impaired the expression of FOXP3. Interestingly, the histone methyltransferase MLL4 binds to this upstream regulatory region and remotely regulates the H3K4 methylation at the promoter and intronic enhancer regions *via* long-distance chromatin looping (**Figure 4**), which exemplifies that a chromatin modification enzyme can modify a chromatin region from a distant binding site through long-distance chromatin interactions. The differentiation of T cells is regulated by not only transcription factors, but also by cytokines. By applying ChIA-PET, Li et al. found that IL-2 activated STAT5, which then regulates the chromatin looping between super-enhancers and promoter of *Il2ra* gene (147).

Mutations in key transcription factors may hamper the differentiation of corresponding T cells, leading to diseases caused by abnormal immune activity. For example, a M370I mutation in FOXP3, identified in an IPEX (immune dysregulation polyendocrinopathy enteropathy X-linked) patient, led to increased chromatin interaction and de-repression of the Th2 cytokine locus in Treg cells in mutation-recapitulated mice as revealed by H3K27ac HiChIP assays (148). These abnormal Treg cells were unable to suppress extrinsic Th2 cells and the mice developed an autoimmune syndrome consistent with an unrestrained T helper type 2 (Th2) immune response. In addition to gene coding regions, mutations in regulatory elements may also alter protein expression and result in the onset of immune diseases. Methods for untangling 3D chromatin structure, such as HiChIP and Hi-C, have been applied to investigate the connection between alternation of the regulome and diseases (49, 79, 149). For example, by applying HiChIP to T cells from type 1 diabetes patients or mouse models, Fasolino et al. discovered the formation of chromatin loops between enhancers and promoters at diabetes risk-conferring loci, indicating that genetic variation leading to the alternation of chromatin structure and gene expression may result in the pathogenesis of autoimmune disorders (150). Widespread alternations in TAD boundary and intra-TAD chromatin interactions were found in T cells of T cell acute lymphoblastic leukemia (T-ALL) patients using Hi-C assays (151). These studies suggest that 3D chromatin structure may critically contribute to the pathogenesis of human diseases and elucidation of the structural changes and their regulation may provide novel insights into the diseases.

## Future Perspectives

In this review, we summarized the approaches for elucidating 3D genome organization, with emphasis on their applications in the immune system. Studies using these tools have advanced our understanding of how genome architecture such as TADs and A/B compartments contributes to the development, differentiation and function of various immune cells. However, many techniques need further improvements in sensitivity and resolution and decreasing background noise. Hi-C related assays could provide high resolution data, which may even reveal promoter-enhancer interactions; however, the sequencing cost is prohibitory to reach its full capacity. Since promoter-enhancer interactions are critical to cell-specific gene expression, highly sensitive and less costly methods for this purpose will be instrumental for elucidating this aspect of chromatin looping. In this regard, TrAC-looping is a method of choice, which specifically reveals interactions among chromatin regulatory elements and thus requires only very modest sequencing depth to cover the entire genome. However, the current TrAC-looping protocol requires 50 to 100 million cells for one experiment, which may be prohibitory for many types of primary cells or clinical samples. A more sensitive TrAC-looping protocol requiring fewer cells is highly demanded.

Genome organization data from bulk cells provides average images of genome architecture of many cells. More studies are demanded to address unique features of individual cells and elucidate how the cellular heterogeneity impacts cellular differentiation and function. Current single cell Hi-C and Dip-C assays suffer from limitations including low efficiency and resolution. To study the genome organization at a single cell level, more versatile approaches are required. For imaging-based approaches, improvements in throughput and resolution should be made to visualize the dynamics of chromatin structure in single cell. With new technical improvement and development, genome architecture, particularly promoter-enhancer interactions, can be analyzed using rare primary cells or patient samples, which will provide more insights into the contribution and mechanisms of chromatin looping in the normal development of immune cells and pathogenic immune disorders.

## Author Contributions

SL and KZ conceived the review idea and wrote the manuscript. All authors contributed to the article and approved the submitted version.

## Acknowledgments

The research in the authors’ laboratory is supported by Division of Intramural Research, National Heart, Lung, and Blood Institute, National Institutes of Health.
